# Reduction of greenhouse gas emissions using the endoscope with a light-emitting diode light source

**DOI:** 10.1055/a-2733-9780

**Published:** 2025-11-06

**Authors:** Katsuya Kikuchi, Tomonori Yano, Yoshikazu Hayashi, Yuji Ino, Takashi Ueno, Satoshi Ozawa, Kentaro Sugano

**Affiliations:** 1 34778 Endoscopy Systems Div. Medical Systems Business Div., FUJIFILM Corporation, Tokyo, Japan; 2 12838 Department of Medicine, Division of Gastroenterology, Jichi Medical University, Shimotsuke, Japan; 3 34778 Medical Systems Research & Development Center, Medical Systems Business Div., FUJIFILM Corporation, Kanagawa, Japan

**Keywords:** Endoscopy Upper GI Tract, Diagnosis and imaging (LCI, BLI, NBI), Endoscopy Lower GI Tract, Small bowel endoscopy

## Abstract

**Background and study aims:**

Light-emitting diodes are being developed as a light source for an endoscope system instead of xenon lamps, contributing to improved diagnostic capability. This system also has the advantage of consuming less power than a conventional endoscope system using xenon lamps. Power consumption associated with endoscopy is an important factor in reducing greenhouse gas emissions. This study estimated the reduction in greenhouse gas emissions by using a light-emitting diode endoscope system.

**Methods:**

We calculated the power consumption and carbon dioxide emission reduction of the light-emitting diode endoscope system in comparison with a conventional endoscope system using a xenon light source. Calculations were based on actual data regarding operating time and the annual number of endoscopic procedures at Jichi Medical University Hospital. Estimates were also extended to a nationwide scale.

**Results:**

If each type of endoscope system were used for all endoscopic procedures, the light-emitting diode endoscope system would reduce power consumption by 49% compared with the xenon endoscope system, resulting in a total power reduction of 838.7kWh, equivalent to 394.2 kg of carbon dioxide emissions. In Japan, the total power consumption would be reduced by 53%, corresponding to a total power reduction of 428,628.8 kWh, equivalent to 201,455.5 kg of carbon dioxide emissions.

**Conclusions:**

We estimated the effect of reducing carbon dioxide emissions using the light-emitting diode endoscope system. Wider adoption of the light-emitting diode endoscope system is expected to contribute to usefulness of endoscopic diagnosis and reduction in environmental impact.

## Introduction

The average global temperature rose above 1.45°C in 2023, reaching a new record high. Climate change poses serious threats to human health and life 1 . Greenhouse gases (GHG) are major contributors to climate change.

In the medical field, GHG emissions account for 4.6% of the world’s total, and their impact on global warming has been widely recognized. In hospital environments, the main departments that emit GHG are operating theaters, intensive care units, and endoscopy suites, which are also among the top three departments generating the most harmful medical waste 2 .

Endoscopy-related academic societies have emphasized the importance of initiatives to reduce the environmental impact of endoscopy and have proposed specific measures to achieve this goal 3 4 5 .

In addition to waste associated with endoscopy, the power consumption involved is also a crucial factor in reducing GHG emissions. Gayam 6 analyzed power consumption of endoscopy-related units and found that the endoscope system accounted for a large proportion of the total power consumption, along with that of the washing machines.

Fujifilm Corporation (Tokyo, Japan) developed a new endoscope system that uses a light-emitting diode (LED) light source instead of a xenon (Xe) light source. An endoscope system (ELUXEO) with four LEDs has been marketed in the West since 2017 and in Japan since 2020 7 8 . This system has the advantage of consuming less power than a conventional endoscope system with the Xe light source. Furthermore, LED light sources are considerably more durable than Xe ones.

In this study, we estimated the reduction effect of GHG emissions using an LED-based endoscope system compared with a conventional endoscope system using a Xe light source.

## Methods

To calculate power consumption during endoscopic procedures, data were collected on the operating time of endoscope systems conducted at Jichi Medical University Hospital. The endoscopic procedures included in this study are esophagogastroduodenoscopy (EGD), colonoscopy (CS), endoscopic retrograde cholangiopancreatography (ERCP), endoscopic submucosal dissection (ESD), and double-balloon enteroscopy (DBE). EGD, CS, and ERCP are routinely performed and a large number of the procedures are performed daily. As a result, an endoscope system remains operational during patient transitions between examinations. In practice, during a procedure, a processor, a light source, and an air/water pump are turned on, whereas during patient transitions, the light source and the air/water pump are turned off. Therefore, both the procedure and standby time during patient transitions were collected. For ESD and DBE, the endoscope system is operated only during each procedure; therefore, only the procedure time was collected.

Power consumption specific to endoscope systems was calculated using the design values for the ELUXEO system with the LED light source and an endoscope system with the Xe light source (Advancia, Fujifilm, Tokyo, Japan) under operating conditions during both procedure and standby times. Power consumption used by the endoscope system was calculated as the product of its power consumption rate and the corresponding operating time. Therefore, power consumption per procedure for EGS, CS, and ERCP was calculated as the sum of the power consumption during the procedure and standby times. For ESD and DBE, power consumption per procedure was calculated based only on procedure time. Annual power consumption was calculated by multiplying power consumption per procedure by the annual number of procedures.

An outcome of this study was to compare power consumption of the endoscope system using a Xe light source with that using an LED light source under actual clinical conditions.

Power consumption data per procedure and annual power consumption data were analyzed using the average and 95% confidence intervals (CIs). A comparison of power consumption between the Xe endoscope system and the LED endoscope system was evaluated based on these averages and 95% CIs. A sensitivity analysis also was included in the comparison.

Power consumption was converted to CO _2_ emissions, using a conversion factor of 0.47 (kg- CO _2_ /kWh) based on the figure published by the Ministry of Economy, Trade and Industry in 2024 9 .

The reduction effect on a national basis also was estimated. This estimation was performed by multiplying average power consumption per procedure by the number of procedures, which was taken from a Japanese procedure database 10 .

This study was exempt from ethical approval according to the Japanese Ethical Guidelines for Medical and Biological Research Involving Human Subjects 11 .

## Results

Operating time data for EGD, CS, and ERCP were prospectively collected between December 16, 2024 and December 24, 2024. Operating time data for ESD for early gastric cancer were collected from procedures conducted in 2023, those of ESD for colorectal neoplasia were collected in 2024, and those of DBE were collected in 2022, all of which were stored in a clinical database at Jichi Medical University Hospital. Data for which accurate examination times were registered were selected. The number of examinations in each category during the 3 years were similar.

Data used for analysis included 119, 53, and 14 consecutive cases of EGD, CS, and ERCP, respectively. ESD for early gastric cancer and DBE datasets comprised 214 and 538 consecutive cases, respectively. For ESD of colorectal neoplasia, 24 cases were analyzed, with one case extracted from every four consecutive procedures.

DBE, ESD, and ERCP were all performed under sedation. EGD and CS were mostly performed without sedation.

Table 1 presents power consumption values specific to both endoscope systems. The LED endoscope system reduced power consumption during the procedure by 38% compared with the Xe endoscope system.

**Table:** **Table 1** Power consumption of endoscope systems.

**Endoscope system**	**Power consumption during procedure (W)**	**Power consumption during standby (W)**
Advancia (Xe)	374.8	57.4
ELUXEO (LED)	141.4	80.6
Advancia (Xe) the endoscope system with the xenon lamp; ELUXEO (LED), the endoscope system with the light-emitting diode. During the procedure, a processor, a light source, and an air/water pump are turned on. During standby, associated with changing patients, a light source and the air/water pump are turned off.		

Table 2 shows operating time and power consumption per procedure. Operating time for EGD, CS, and ERCP includes standby time associated with changing transitions. Standby times for EGD, CS, and ERCP were fixed values corresponding to the average of the collected data: 11.7, 15.4, and 27.4 minutes, respectively. Power consumption per procedure was calculated as the product of procedure time and power consumption value of the endoscope systems listed in Table 1 .

**Table:** **Table 2** Operating time and power consumption per procedure.

**Procedure items**	**Average operating time (min)**		**Power consumption (Wh)**			
	**Mean**	**95% CI**	**Xe**		**LED**	
			**Mean**	**95% CI**	**Mean**	**95% CI**
EGD ^*^	18.9	18.3–19.4	54.4	51.1–57.9	32.4	31.2–33.8
CS ^*^	38.8	35.8–42.0	156.2	138.8–175.8	75.1	68.3–82.6
ERCP ^*^	55.7	47.3–65.5	193.4	145.6–257.0	102.1	83.1–125.4
ESD for early gastric cancer ^†^	79.8	74.0–86.0	498.3	462.1–537.4	188	174.3–201.7
ESD for colorectal neoplasia ^†^	116.2	88.4–152.6	725.8	552.4–953.5	273.8	208.4–359.7
DBE ^†^	57.8	55.4–60.3	360.8	345.9–376.4	136.1	130.5–142.0
CI, confidence interval; CS, colonoscopy; DBE, double-balloon enteroscopy; EGD, esophagogastroduodenoscopy; ERCP, endoscopic retrograde cholangiopancreatography; ESD, endoscopic submucosal dissection; LED, light-emitting diode endoscope system; Xe, xenon endoscope system. The formula for calculating power consumption is as follows: ^*^ Power consumption per procedure (Wh) = {procedure time (min) x power consumption during procedure (W) + standby time (min) x power consumption during standby (W)}/60. ^†^ Power consumption per procedure (Wh) = {procedure time (min) x power consumption during procedure (W)}/60.						

Because data for operating time and power consumption for each procedure showed a log-normal distribution trend, log-transformed data were used to calculate the mean and 95% CIs. Results are presented as geometric means and corresponding 95% CIs.

For all procedure types, power consumption per procedure using the LED endoscope system was significantly lower than that for the Xe endoscope system. The upper limit of the 95% CIs for the LED endoscope system was lower than the lower limit of those for the Xe endoscope system, indicating a statistically significant difference.

Table 3 presents annual power consumption based on annual number of procedures at Jichi Medical University Hospital. Power consumption was calculated by assuming that all procedures were performed using either the Xe or LED endoscope systems. Average total power consumption of the LED endoscope system was 49% of that of the Xe endoscope system.

**Table:** **Table 3** Annual power consumption at Jichi Medical University Hospital.

**Procedure items**	**Number of annual procedures (FY2022)**	Annual power consumption (kWh) ^*^			
		**Xe**		**LED**	
		**Mean**	**95% CI**	**Mean**	**95% CI**
EGD	7,151	388.7	365.1–413.8	231.9	222.9–241.4
CS	4,437	693.1	615.9–779.9	333.3	303.1–366.5
ERCP	724	140.1	105.4–186.1	73.9	60.2–90.9
ESD for early gastric cancer	281	140	129.9–151.9	52.8	49.0–57.0
ESD for colorectal neoplasia	105	76.2	58.0–100.1	28.8	21.9–37.8
DBE	540	194.8	186.8–203.2	73.5	70.5–76.7
Total	13,238	1632.9	-	794.2	-
CS, colonoscopy; DBE, double-balloon enteroscopy; EGD, esophagogastroduodenoscopy; ERCP, endoscopic retrograde cholangiopancreatography; ESD, endoscopic submucosal dissection; FY, fiscal year; LED, light-emitting diode endoscope system; Xe, xenon endoscope system. The formula for calculating annual power consumption is as follows: ^*^ Annual power consumption (kWh) = {Power consumption per procedure (Wh) x Number of annual procedures}/1000.					

Table 4 summarizes annual power consumption reduction between the Xe and LED endoscope systems. The reduction is presented as a base-case scenario for the difference in average values, as the best-case scenario for the difference in the upper limit of the 95% CIs, and as the worst-case scenario for the difference in the lower limit of the 95% CIs. In the base-case scenario, 838.7 kWh of power was saved, equivalent to 394.2 kg of CO _2_ emissions.

**Table:** **Table 4** Annual power reduction at Jichi Medical University Hospital.

**Procedure items**	**Annual power reduction (kWh)**		
	Base ^*^	** Best ^†^**	** Worst ^‡^**
EGD	156.8	172.4	142.2
CS	359.8	413.4	312.8
ERCP	66.2	95.2	45.2
ESD for early gastric cancer	87.2	94.9	80.9
ESD for colorectal neoplasia	47.4	62.3	36.1
DBE	121.3	126.5	116.3
Total	838.7	964.7	733.5
EGD, esophagogastroduodenoscopy; CS, colonoscopy; ERCP, endoscopic retrograde cholangiopancreatography; ESD, endoscopic submucosal dissection; DBE, double-balloon enteroscopy. The formula for calculating annual power reduction is as follows: ^*^ Difference in average values between the Xe and LED endoscope systems. ^†^ Difference in upper limits of the 95% confidence intervals between the Xe and LED endoscope systems. ^‡^ Difference in lower limits of the 95% confidence intervals between the Xe and LED endoscope systems.			

The estimated reduction effect on a domestic basis was calculated using the average power consumption per procedure at Jichi Medical University Hospital and the number of domestic procedures reported in Japan’s National Database (NDB) for the fiscal year 2022 10 , focusing on endoscopies with a high number of procedures, such as EGD, CS, and ESD. Assuming all endoscopic procedures were performed using the LED endoscope system, total power consumption would be reduced to 53% of that with the Xe endoscope system, resulting in a power savings of 428,628.8 kWh ( Table 5 ). That amount of power saved was equivalent to 201,455.5 kg of CO _2_ emissions, comparable to driving 513,018 miles using an average gasoline-powered passenger vehicle 12 . Differences in power consumption reduction rates between Jichi Medical University Hospital and the domestic basis are due to differences in procedure items evaluated.

**Table:** **Table 5** Annual power consumption and power reduction in Japan.

**Procedure items**	**Number of annual procedures (FY2022)**	Annual power consumption (kWh) ^*^		** Annual power reduction (kWh) ^†^**
		**Xe**	**LED**	
EGD	7,877,146	428,516.7	255,219.5	173,297.2
CS	2,793,712	436,377.8	209,807.8	226,570.0
ESD for early gastric cancer	49,399	24,615.5	9,287.0	15,328.5
ESD for colorectal neoplasia	29,719	21,570.1	8,137.1	13,433.0
Total	10,749,976	911,080.1	482,451.4	428,628.8
CS, colonoscopy; EGD, esophagogastroduodenoscopy; ESD, endoscopic submucosal dissection; FY, fiscal year; LED, light-emitting diode endoscope system; Xe, xenon endoscope system. The formula for calculating annual power consumption and reduction is as follows: ^*^ Annual power consumption (kWh) = {Power consumption per procedure (Wh) x Number of annual procedures}/1000. ^†^ Annual power reduction (kWh) = (Annual power consumption of the Xe endoscope system) - (Annual power consumption of the LED endoscope system).				

## Discussion

The effect of the LED endoscope system in reducing CO _2_ emissions has been demonstrated using data from real-world endoscopic procedures. The results showed that the amount of power consumed by the LED endoscope system at Jichi Medical University Hospital was almost half that of the Xe endoscope system, indicating a significant reduction in CO _2_ emissions. This provides the first evidence supporting the superiority of LED systems, as described in the position statement by the European Society of Gastrointestinal Endoscopy and the European Society of Gastroenterology and Endoscopy Nurses and Associates 3 .

Several studies have reported on the environmental impact of endoscopic devices 6 , washing machines 6 , and histopathological diagnoses 13 related to endoscopic procedures. This study analyzed the effect of reducing CO _2_ emissions using an endoscope system with an improved light source.

Reports have also highlighted the impact of avoiding unnecessary procedures on the environmental burden based on accurate diagnosis. Yusuf et al. estimated reductions in GHG emissions by evaluating the number of medical procedures avoided during CS screening 14 . Cho et al. estimated reduction in CO _2_ emissions and medical costs resulting from the high diagnostic performance of narrowband imaging observation in diagnosing gastrointestinal epithelial hyperplasia 15 . Ueda et al. proposed a strategy for reducing GHG emissions and maintaining high-quality patient care by reducing unnecessary surveillance endoscopy and biopsy through adoption of accurate methods for endoscopic diagnosis, such as image-enhanced endoscopy (IEE) and artificial intelligence 16 .

The LED endoscope system was equipped with IEE functions for blue light imaging (BLI) and linked color imaging (LCI). Reports have shown that BLI and LCI have significantly higher detection and differentiation capabilities for upper gastrointestinal tumors and colorectal neoplasms than white light imaging 15 16 17 18 19 . Similar findings were reported in other countries such as China and Brazil 20 21 . These capabilities facilitate targeted rather than random biopsies, thus contributing to a reduction in the number of biopsy samples.

By adopting the LED endoscope system, we can expect to reduce power consumption, thereby decreasing environmental burden owing to improvement in diagnostic performance.

In this study, we investigated power consumption of endoscope systems used in real clinical settings at a hospital. The investigation did not include consideration of the supply chain associated with product manufacturing. The two types of endoscope systems examined in this study differ only in their light source units for illumination. The environmental impact of the endoscope systems, excluding the light source units, is considered equivalent throughout their entire lifecycle.

In clinical hospital environments, in addition to power consumption, replacement and disposal of light source units due to their lifespan also pose challenges. Regarding the lifespan of light source units, the LED light source is superior to the Xe light source in terms of its lifecycle. The LED light source has a lifespan equivalent to that of the endoscope system, whereas the Xe light source has a lifespan of 500 hours 22 23 . However, the environmental impact associated with replacement and disposal of Xe light sources due to their short life span accounts for only a small percentage of annual power consumption under the usage conditions at Jichi Medical University Hospital. Therefore, this study only considered power consumption as the measurement target.

This study has limitations. The reduction effect on a domestic basis was an approximate prediction result extrapolated using the conditions of the endoscopy suites at a single university hospital. Another limitation is that power consumption of Olympus endoscope systems has not been evaluated.

## Conclusions

In conclusion, the effect of reducing CO _2_ emissions using the LED endoscope system has been demonstrated. Wider adoption of the LED endoscope system will contribute to usefulness of endoscopic diagnosis and reduce the environmental impact.
